# Evaluation of prognostic value of vascular endothelial growth factor and endocan in non-small cell lung cancer

**DOI:** 10.55730/1300-0144.6100

**Published:** 2025-10-05

**Authors:** Mesut Melih ÖZERCAN, Burcu ANCIN, Yiğit YILMAZ, Ahmet MÜDERRİSOĞLU, Serkan UYSAL, Ulaş KUMBASAR, Zeynep SARIBAŞ, Erkan DİKMEN, Rıza DOĞAN

**Affiliations:** 1 Department of Thoracic Surgery, Faculty of Medicine, Kırıkkale University, Kırıkkale, Turkiye; 2 Department of Thoracic Surgery, Burdur State Hospital, Burdur, Turkiye; 3 Department of Thoracic Surgery, Faculty of Medicine, Hacettepe University, Ankara, Turkiye; 4 Department of Pharmacology, Faculty of Medicine, Kırıkkale University, Kırıkkale, Turkiye; 5 Department of Medical Microbiology, Faculty of Medicine, Hacettepe University, Ankara, Turkiye; 6 Emeritus, Department of Thoracic Surgery, Faculty of Medicine, Hacettepe University, Ankara, Turkiye

**Keywords:** Endocan, non-small cell lung cancer, prognosis, screening, VEGF

## Abstract

**Background/aim:**

No reliable, easily measurable biomarker currently exists for the screening or prognosis of lung cancer. The present study evaluates the potential utility of vascular endothelial growth factor (VEGF) and endocan, which can be readily measured in blood samples, as biomarkers for screening and prognostic assessment in lung cancer.

**Materials and methods:**

Included in the study were 38 patients with early-stage non-small cell lung cancer (NSCLC) and 29 controls. All patients underwent surgical intervention and were monitored for 1 month postoperatively. Serum VEGF and endocan levels were measured preoperatively and postoperatively. Clinical characteristics, laboratory data, and histopathological findings were recorded for all participants.

**Results:**

The preoperative VEGF levels of the patients were significantly higher than those of the controls (p = 0.001), while postoperative VEGF levels decreased significantly following surgery (p < 0.001). The endocan levels of the patients and controls were similar preoperatively. Additionally, endocan levels were significantly increased after the surgery in the patient group (p < 0.001). A comparison of VEGF and endocan levels according to TNM staging and tumor histopathology revealed no significant differences.

**Conclusion:**

VEGF can serve as a potential biomarker for screening and prognostic assessment in early-stage NSCLC. In contrast, endocan did not demonstrate utility for such clinical purposes. VEGF may also be considered for the evaluation of treatment efficacy following surgical intervention.

## 1. Introduction

Globally, cancer remains the second leading cause of death after ischemic heart disease, with lung cancer being the most common form in both sexes. In 2022, lung cancer accounted for 12.4% (2.8 million) of new cancer diagnoses, and 18.7% (1.8 million) of cancer-related deaths. These numbers are expected to rise in the near future [1].

There are four histopathological types of lung cancer: adenocarcinoma, squamous cell carcinoma (SCC), large cell carcinoma, and small cell carcinoma [2]. Non-small cell lung cancer (NSCLC) accounts for 85% of all lung cancer cases [3]. Surgery, chemotherapy, radiotherapy, and immunotherapy are the primary interventions for the treatment of NSCLC, with the choice of treatment depending on tumor stage (grade, lymph node involvement, metastasis) and histopathological type, as well as such specific biomarkers as epidermal growth factor receptor (EGFR) status. Early diagnosis, treatment, and close surveillance are known to be essential for better prognosis. For this reason, the identification of reliable biomarkers for cancer monitoring remains a priority [4].

Tumor angiogenesis has emerged as a promising direction in biomarker research, as a fundamental process that supports tumor growth and progression. In the absence of adequate angiogenesis, a tumor can only reach 1 mm^3^ in volume through cell proliferation [5]. Cancer cells in hypoxic environments secrete vascular endothelial growth factor (VEGF) and overexpress VEGF receptor 2 to promote angiogenesis [6]. Such increased levels of angiogenesis allow the tumor to grow, and tumor angiogenesis, referred to as angiogenic switch, is a key factor driving progression to malignancy [7]. Angiogenesis resulting from elevated VEGF levels (typical range in serum: 62–707 pg / mL) has been identified as a key factor in malignancy development, and has prompted studies into VEGF inhibitors as potential cancer treatments. Increased VEGF levels have been identified in patients with NSCLC [8], and this had led to the inclusion of such VEGF inhibitors as bevacizumab, ramucirumab, and nintedanib in adjuvant treatment strategies for NSCLC in clinical practice [4]. There have also been studies promoting the use of VEGF as a prognostic indicator for NSCLC, given its association with low survival in NSCLC patients [9]. However, these are contrasted by other studies in the literature reporting no significant association between VEGF level and NSCLC prognosis [10]. There is, therefore, a lack of conclusive evidence supporting the use of VEGF as a prognostic indicator for NSCLC [11].

Endocan (known formerly as endothelial cell specific molecule-1) is a soluble dermatan sulphate proteoglycan that is primarily secreted by lung endothelial cells [12]. A number of previous studies have suggested that endocan may contribute to tumor development through its effects on such key cellular processes as differentiation, migration, and adhesion [13,14]. Moreover, endocan overexpression in cancer cells has been demonstrated [15]. Grigoriu et al. reported considerably elevated levels of endocan (typical range in serum: 300–1200 pg / mL) in lung tumor tissue when compared to healthy lung tissue, and suggested that endocan may correlate with adverse prognosis in lung cancer patients [16]. VEGF is known to increase the production or secretion of endocan [17]. Based on this knowledge, there have been studies investigating the relationship between endocan and tumor vascularization, reporting an association between endocan and microvascularization level in some cancers, including colorectal and hepatocellular carcinomas [18,19]. In another study, elevated endocan levels were reported to be directly correlated with tumor size in lung cancer patients during the pretreatment phase [20]. The results of these studies suggest that endocan may have potential for the screening or prognostic evaluation of lung cancer.

In this study, we hypothesize that VEGF and endocan serum levels may serve as novel biomarkers for the screening and prognostic indication of early-stage NSCLC. To test this theory, we measured VEGF and endocan levels in early-stage NSCLC patients before and after surgery, and compared the results with those of a control group. There have been no studies in the literature to date evaluating serum VEGF and endocan levels concurrently in patients with early-stage NSCLC.

## 2. Materials and methods

This study was conducted in accordance with national regulations and institutional policies, as well as the tenets of the Helsinki Declaration. The study protocol was approved by the institutional review board of our institution (Hacettepe University Ethics Board, 2020/09-37), and informed consent was obtained from all participants.

Included in this prospective study were patients recruited from the Department of Thoracic Surgery, Hacettepe University Faculty of Medicine, between June 2020 and January 2021, with 38 patients scheduled for surgical resection of NSCLC recruited as the patient group and 29 patients hospitalized for non-malignant lung conditions as the control group. Excluded from the study were patients with a secondary cancer, any organ insufficiency or failure, or under 18 years of age, as well as pregnant women. The age, sex, body mass index, smoking-concurrent disease status, and laboratory values (complete blood counts-including total leukocyte, neutrophil, and lymphocyte counts-and C-reactive protein [CRP] levels) of all participants in the patient group were recorded before and after the surgery, and at the time of the hospitalization in the control group. Details of TNM staging (T: tumor size, N: lymph node involvement, M: metastasis), chemotherapeutic regimen, and surgical procedures were documented for the patient cohort.

A single blood sample was obtained from the control group upon hospitalization for the assessment of VEGF and endocan. In the patient group, blood samples were taken at four time points: preoperatively (at admission, before surgery), and on postoperative days 1, 7, and 30. Serum samples were separated from blood and stored at −80 °C until the time of analysis. VEGF and endocan levels were measured using ELISA kits according to the manufacturer’s instructions (Cloud Clone Corp, Texas, USA).

The required sample size was calculated using PASS 11 software (NCSS, Utah, USA), calculated with a type I error probability of 0.05 and power of 80%. For a study with at least one control per case, the calculated number was 27 for each group.

IBM SPSS Statistics (Version 29.0. Armonk, NY: IBM Corp, 2023) was used for the statistical analyses. Descriptive statistics were presented as mean ± standard deviation (95% confidence intervals) for the results of the parametric tests, and as median (range) for the results of the non-parametric tests. The normality of distributed data was assessed using the Shapiro-Wilk test. For normally distributed data, comparisons were made using the t-test, one-way ANOVA, and post-hoc Tukey’s test, while Mann-Whitney U, Kruskal-Wallis, and post-hoc Dunn’s tests were used for non-normally distributed data. Repeated measures ANOVA were used for normally distributed paired data, and the Friedman test for non-normally distributed paired data, respectively. Correlations between clinical and laboratory parameters and VEGF and endocan levels were analyzed using Spearman’s rank correlation coefficient. P < 0.05 was accepted as statistically significant.

## 3. Results

The ratios of males in the patient and control groups were 81% (n=31) and 52% (n=15), respectively. The mean age was 61.1 ± 9.2 years in the patient group and 41.9 ± 16.4 in the control group. Among the patients, four (10.5%) were treated with neoadjuvant chemotherapy, 31 (81.6%) were smokers, seven (18.4%) had type II diabetes mellitus, eight (21%) had concurrent pulmonary disease (5 COPD, 2 asthma, 1 sarcoidosis), and 17 (44.7%) had cardiovascular comorbidities such as hypertension, coronary artery disease, hyperlipidemia, or arrhythmia. Preoperative VEGF levels were significantly higher in the patients than in the controls (p = 0.001). An evaluation of VEGF levels recorded before surgery and on days 1, 7 and 30 following surgery revealed that VEGF levels in the NSCLC patient group decreased significantly over time (p < 0.001). Notably, the preoperative endocan levels of the patient group were similar to those of the controls (p = 0.16), but increased over time (p<0.001). These results are summarized in Table 1 and Figure.

A comparison of the biomarker levels of the male and female patients at each time point revealed a significant difference only for endocan on the 30^th^ postoperative day, when the median endocan level on day 30 was 789.9 pg / mL (range 198.2–2061.2) in males and 330.9 (244.9–587.3) in females (p= 0.022). No significant differences in VEGF or endocan levels were noted between males and females in the control group.

Only age was found to be positively correlated with preoperative VEGF (r = 0.397, p = 0.014) and endocan (r= 0.363, p= 0.025) levels among the evaluated clinical characteristics and laboratory findings of the patient group. While no correlation was observed between age and endocan levels in the control group, it was interesting to note that VEGF was negatively correlated with age (r = −0.444, p = 0.016). Furthermore, no associations were identified between any clinical variables or laboratory data and postoperative day 30 VEGF or endocan levels in the patient group. The results are presented in Supplementary Tables 1 and 2.

Most of the patients had early-stage NSCLC, with T scores of T1 in 17 patients, T2 in 13 patients, and T3 in eight patients, and five patients had visceral pleural invasion. The N scores were N0 for 31 patients and N1 for seven patients. No metastasis was detected in any of the patients (M0 for all 38 patients), and pleural invasion status did not differ when the patients were grouped according to TNM staging. Group comparisons based on T-scores and N-scores are presented in Tables 2 and 3, respectively.

A histopathological evaluation of resected tumor tissues showed that 20 (52.6%) patients had adenocarcinoma, 10 (26.3%) had SCC, and 8 (21%) had neuroendocrine tumors. Postoperative VEGF levels on day 7, postoperative endocan levels on day 1, and preoperative CRP levels differed significantly between the groups according to histopathological tumor classification (p values: 0.034, 0.041, and 0.045, respectively). Post-hoc analysis revealed significantly higher VEGF levels in patients with SCC compared to those with NET on postoperative day 7 (adjusted p = 0.038). Conversely, endocan levels on postoperative day 1 were higher in patients with NET than in those with SCC (adjusted p = 0.035). Additionally, the elevation of preoperative CRP levels was more pronounced in patients with SCC than in those with NET (adjusted p = 0.043). These findings are summarized in Table 4.

Analyses of VEGF levels, endocan levels, clinical characteristics, and laboratory findings between the patient groups classified based on neoadjuvant chemotherapy receiving status, smoking status, presence of type II diabetes mellitus, secondary pulmonary disease, and cardiovascular disease revealed no statistically significant differences, other than in endocan levels on postoperative day 30, which were significantly higher in the smoking group than in the non-smoking group (p = 0.043). Detailed results are presented in Supplementary Table 3. Furthermore, no correlation of postoperative serum VEGF and endocan levels was identified with any inflammatory markers (CRP, white blood cell-neutrophile-lymphocyte counts, neutrophile to lymphocyte ratio).

## 4. Discussion

In our study’s primary findings, preoperative serum VEGF levels were noted to be elevated in early-stage NSCLC patients when compared to the controls, but decreased considerably following surgical resection and remained low throughout the 1-month postoperative follow-up. In contrast, the serum endocan levels of the patients and controls were similar at baseline, but increased significantly after surgery and remained above preoperative levels at 1 month follow-up (Table 1, Figure). Postoperative levels of VEGF and endocan were correlated with the histological type of the tumor, while preoperative levels were linked to patient age (Table 4, Supplementary Table 1). Additionally, postoperative endocan levels were found to be associated with smoking status in NSCLC patients (Supplementary Table 3).

Lung cancer is the most common form of the disease and is the leading cause of cancer-related death [1]. Accordingly, there is a need for reliable, specific, and easily testable lung cancer biomarkers to support screening and prognosis evaluations. The currently available biomarkers, including carcinoembryonic antigen, Kirsten rat sarcoma viral oncogene (*KRAS*), and *EGFR* mutations, are either not specific to lung cancer, making them unreliable for diagnosis, or require genetic testing, which is not widely available and there may be a long wait for results [21]. Consequently, there is a pressing need for a reliable and easily testable biomarker that can support clinical lung cancer screening and monitoring. VEGF and endocan are reasonably simple and rapid to assess; however, evidence of their reliability in lung cancer screening and surveillance applications remains ambiguous [22].

Solid tumor growth and metastasis are known to be dependent on angiogenesis, which requires VEGF [5]. Based on this understanding, VEGF inhibitors such as bevacizumab are included in some current lung cancer treatment protocols and have been shown to improve survival rates among NSCLC patients [4,8]. Immunotherapy has emerged as a promising therapeutic modality for lung cancer, and for various other malignancies. A recent study suggests that VEGF levels may serve as a predictive biomarker for the identification of NSCLC patients who are likely to benefit from the inclusion of bevacizumab in their immunotherapy regimens [11]. In a study of 451 patients, however, serum VEGF levels were demonstrated to have low ability in differentiating between NSCLC and benign lung diseases [23]. Similar to the findings of studies by Lai et al. and Kishiro et al. reporting considerably higher levels of serum VEGF in primary NSCLC patients than in controls without cancer, significantly increased serum VEGF levels in NSCLC patients were noted also in the present study [24,25]. In the present study, preoperative VEGF levels in the NSCLC patients were significantly higher than in the controls, and declined continuously throughout the first postoperative week to reach a level similar to that of the controls, after which they plateaued for 1 month. This finding is consistent with results reported previously by Lai et al. and Rather et al. [24,26], both of which reported decreased VEGF levels after surgery, although Rather et al. reported observing this decrease at 1 month following surgery [24,26]. Our findings regarding VEGF support its use as a biomarker for NSCLC screening and as a prognostic indicator for the detection of response to treatment, surveillance, and relapse in early-stage NSCLC patients undergoing surgery.

VEGF’s inducing effect on endocan secretion has been proven [17], and the promotion of a more aggressive cancer cell phenotype in NSCLC by endocan has been identified [27]. In further studies, serum endocan level has been associated with tumor size and poor prognosis in primary lung cancer patients, highlighting its potential as a prognostic marker in lung cancer [16,20]. In contrast to the findings of the mentioned studies, our study showed similar levels of endocan between the NSCLC patients and controls without lung cancer, as well as significantly increased endocan levels 1 month after surgery when compared to preoperative levels in the NSCLC patients. Lung cancer is often diagnosed at later stages, with only 21% of patients being eligible for surgery at the time of diagnosis.^1^ For this reason, most clinical studies seek to identify a biomarker for lung cancer based on analyses of later-stage lung cancer patients. Unlike most previous studies, the majority of patients in the present study had early-stage NSCLC. Our finding that endocan is unsuitable as a prognostic indicator of NSCLC can be attributed to the predominance of early-stage cancer patients in the study. It has been well established that inflammation increases serum endocan levels [17]. The observed increase in endocan levels on the first and seventh postoperative days may therefore be attributable to the increased inflammation following surgery. Endocan levels remained higher than the preoperative level on postoperative day 30, suggesting that inflammatory response persisted for at least 1 month. In fact, the postoperative CRP levels were higher than the preoperative levels, indicating exaggerated inflammation after surgery. This inflammatory surge in endocan reduces its specificity for lung cancer, and hinders its usefulness as a screening or prognostic marker. Accordingly, this study suggests that endocan may be unsuitable for screening NSCLC or for predicting prognosis in early-stage NSCLC.

Tang et al. reported no association of age with VEGF mRNA expression levels in NSCLC [28]. Furthermore, Lu et al. reported a lack of correlation between age and serum endocan level in NSCLC patients [29]. In contrast to the findings of Tang et al. and Lu et al., associations between age and VEGF, as well as endocan levels, were identified in the preoperative period in the present study that were absent in the postoperative period. We thus consider our finding regarding age to be inconclusive. Additionally, since lung cancer is more common in older patients, the ages of the patients were significantly higher than those of the control group, which may affect the comparison of the two groups.

Both Grigoriu et al. and Tang et al. reported no difference between sexes regarding VEGF levels in NSCLC patients [16,28]. Similarly, no significant difference was noted in serum VEGF levels between the male and female NSCLC patients in the present study other than on postoperative day 30, when significantly lower VEGF levels were reported in the female patients. Since the incidence of lung cancer is higher in men than in women [1], our study had a larger number of male patients (81%), which makes the findings related to sex less conclusive.

Shimanuki et al. reported no association between preoperative serum VEGF levels and T score in 63 NSCLC patients in their study who later underwent surgery [30]. In contrast, Kaya et al. reported that NSCLC patients with high T scores (T3–4) had higher levels of serum VEGF than those with low T scores (T1–2) [31]. Our discovery of no correlation between T score and blood VEGF level aligns with the findings reported by Shimanuki et al. [30]. All of the patients in the present study and the study of Shimanuki et al. underwent surgery, while only 30.6% of the patients in Kaya et al.’s study underwent surgery, as most had later-stage, inoperable NSCLC [31]. When examined together with the results of the mentioned studies, our reported relationship of T score with VEGF indicates that VEGF has low sensitivity in differentiating tumor sizes. That said, while VEGF may be considered unsuitable for differentiating T scores in early-stage NSCLC (T1–2), it may be used to differentiate between high T scores (T3–4) and low T scores (T1–2). Grigoriu et al. reported no relationship between T score and serum endocan level, while Scherpereel et al. showed a positive correlation between the two in their study of 50 lung cancer patients [16,20]. Our finding of no association between T score and serum endocan level supports the finding reported by Grigoriu et al. [16].

The mechanism of lymphangiogenesis is thought to be similar to that of angiogenesis, and VEGF is the primary mediator in both processes. Moreover, VEGF can induce the metastatic spread of tumors via lymph vessels. By secreting VEGF, tumor cells induce new lymphatic vessel generation to ensure their spread [32]. Therefore, it was hypothesized that high serum VEGF level could be associated with high N stage in solid tumors, and this hypothesis was investigated accordingly [24]. While Lai et al. reported that serum VEGF levels vary significantly across different N stages in NSCLC patients [24], there have been several other studies reporting no such association [30,33]. Our study revealed similar serum VEGF levels in our patient set when grouped according to the N stage (Table 3), while N score was not associated with serum endocan levels, consistent with the findings previously reported by Grigoriu et al. [16].

Inflammation contributes to the formation of the optimal conditions for tumor development, while tumor intrinsic factors can contribute heightened inflammatory state in cancer patients [34]. Inflammatory markers, WBC, CRP, and NLR show increased inflammation and are induced by similar inflammatory cytokines such as tumor necrosis factor-α and interleukin (IL)-6 [35]. While serum VEGF level in small cell lung cancer patients have been found to correlate with IL-6 and IL-8 [36,37], VEGF has been found to be associated with WBC in NSCLC patients [38]. In contrast, no correlation between serum VEGF and WBC, CRP, or NLR was identified in the present study; and likewise, no correlations were noted between serum endocan and WBC, CRP, or NLR in NSCLC patients. This is the first study in the literature to investigate the association of serum endocan level with WBC, CRP, and NLR in NSCLC patients.

There remains a lack of consensus in the literature on whether an association between histopathological subtypes of NSCLC and VEGF level exists. While some studies have reported no significant relationship between NSCLC subtypes and serum VEGF levels [16,28], others have suggested a potential association. For instance, Imoto et al. reported significantly higher levels of VEGF receptor expression in adenocarcinoma compared to SCC [33], whereas Shimanuki et al. identified significantly lower serum VEGF levels in patients with adenocarcinoma than in those with SCC [30]. In the present study, no significant difference was observed in preoperative serum VEGF levels among patients with adenocarcinoma, SCC, and neuroendocrine tumors. However, significantly higher serum VEGF levels were noted on the seventh postoperative day in patients with SCC than in those with neuroendocrine tumors. Furthermore, on the first postoperative day, serum endocan levels were significantly higher in the patients with neuroendocrine tumors than in those with SCC. On the other hand, preoperative CRP levels were significantly higher in patients with SCC than in patients with neuroendocrine tumors. Among the histological subtypes of NSCLC, SCC is most significantly correlated with smoking, which elevates CRP levels [39,40]. Higher preoperative CRP levels in patients with SCC may reflect heavier smoking this patient group close to surgery compared to other patients.

Duan et al. reported no significant change in serum VEGF levels before and after cisplatin-gemcitabine chemotherapy in patients with NSCLC [41]. Similarly, Song et al. found no significant difference in serum VEGF levels between the pre-treatment and post-treatment periods in NSCLC patients whose disease continued to progress, despite chemotherapy. That said, in the same study, a significant decrease in serum VEGF levels was noted following chemotherapy in patients responded well to treatment [42]. These findings suggest a link between VEGF levels and treatment effectiveness, and this is supported by the present study, in which significantly reduced serum VEGF levels were noted after surgical resection. Taken together, these findings support the consideration of VEGF level as an indicator of treatment effectiveness, whether chemotherapy or surgery. Similar preoperative serum VEGF levels were noted in NSCLC patients who received neoadjuvant chemotherapy to those who did not. Our study can be considered notable as the first to report no association between serum endocan level and neoadjuvant chemotherapy receiving status.

In recognition of the studies indicating elevated serum VEGF and endocan in patients with hypertension or type II DM [43,44], we compared the levels of these two biomarkers between NSCLC patients with and without the stated conditions, revealing no significant differences in either biomarker between the groups. In contrast, a significant difference was noted in the serum endocan levels of patients grouped according to smoking status, which may be a result of the effect of smoking on inflammation, which is known to elevate endocan levels [39].

There are a number of limitations to our study that should be noted. While the statistical power analysis revealed that the number of patients and controls was sufficient to reach a statistical power of 0.8 in a comparison of serum VEGF and endocan levels between the two groups, an analysis of patient subgroups based on TNM staging, histopathological classification, smoking status, chemotherapy regimen, and presence of concurrent disease was performed with smaller subgroups, resulting in a statistical power of less than 0.8. A further limitation related to the different mean age and sex ratios of the groups. Since lung cancers are mostly seen in older males, while benign lung diseases are distributed across all ages and genders, we were unable to recruit participants with similar demographics for each group. Furthermore, our postoperative monitoring of NSCLC patients was limited to 1 month; however, a longer surveillance period is required for the effective evaluation of VEGF and endocan as a marker for the prognosis of early-stage NSCLC. Despite its shortcomings, we believe that this study can be included among the few studies in literature contributing to the efforts to identify reliable and easy-to-test biomarkers for lung cancer, as the leading cause of cancer-related death.

In conclusion, our findings suggest that VEGF may serve as a useful biomarker in early-stage NSCLC, both as an indicator of surgical treatment effectiveness and as a tool for early screening. The results of this study reveal that VEGF levels did not vary across different TNM stages or histological subtypes within the sample. For this reason, clinical applications of VEGF should be limited to evaluations of treatment success and screening for NSCLC. This study further identifies endocan as an inadequate marker for the screening and evaluation of prognosis in NSCLC. We believe that VEGF could eventually be adopted into clinical practice for the prognostic evaluation of NSCLC if the findings of the present study can be corroborated by evidence from future studies, ideally with larger cohorts and longer follow-up periods (at least 6 months).

## Supplementary Information

**Supplementary Table 1:** Correlations of preop VEGF and endocan levels with evaluated clinical characteristics and laboratory findings in patients

	Preop VEGF		Preop Endocan	
	r	p	r	p
**Age**	0.397	**0.014**	0.363	**0.025**
**Smoking (pack-year)**	−0.084	0.617	0.092	0.582
**Tumour Size**	−0.004	0.982	0.074	0.66
**Preop WBC**	−0.206	0.216	−0.05	0.764
**Preop NLR**	0.244	0.14	−0.281	0.087
**Preop CRP**	−0.162	0.333	0.274	0.096

WBC: white blood cell count, NLR: neutrophil to lymphocyte ratio, CRP: C-reactive protein

Statistically significant p values were marked in bold.

**Supplementary Table 2:** Correlations of postop 30^th^ day VEGF and endocan levels with evaluated clinical characteristics and laboratory findings in patients

	Preop VEGF		Preop Endocan	
	r	p	r	p
**Age**	0.305	0.08	0.099	0.576
**Smoking (pack-year)**	−0.133	0.452	−0.055	0.757
**Tumour Size**	−0.136	0.444	−0.024	0.892
**Postop WBC**	−0.156	0.38	0.203	0.25
**Postop NLR**	0.275	0.115	0.204	0.247
**Postop CRP**	−0.134	0.449	−0.04	0.823

WBC: white blood cell count, NLR: neutrophil to lymphocyte ratio, CRP: C-reactive protein

**Supplementary Table 3:** Comparison of endocan levels between the patients grouped as never-smokers, smokers with ≤30 pack-years of smoking, and smokers with >30 pack-years of smoking

	Never-smokers	Smokers	p Value
**Preop endocan level (pg/ml)**	533 (175.5–1595.5)	377.4 (193.1–1573.3)	0.973
**Postop 30** **^th^** ** day endocan level (pg/ml)**	337.7 (301.5–597.2)	803.4 (198.2–2016.2)	**0.033**

Results have been demonstrated as median (range). Statistically significant p values were marked in bold.

## Figures and Tables

**Figure:**
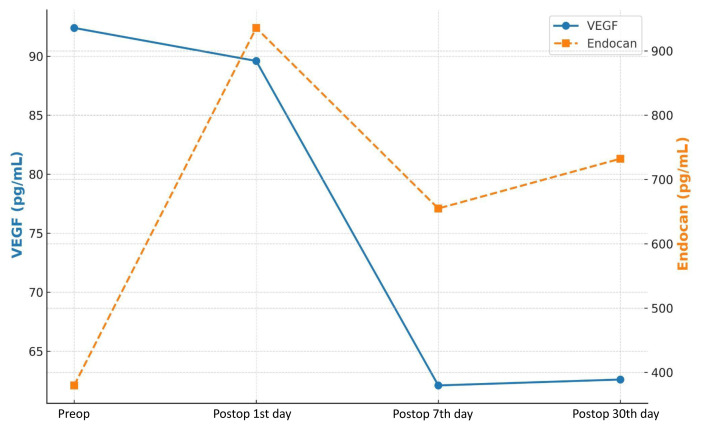
Preop and postop 1^st^–7^th^–30^th^ day levels of VEGF in the patient group were 92.4 (11.2–338.4), 89.6 (0.7–293.5), 62.1 (0.1–353), and 62.6 (0.1–353.7) pg/mL, respectively (p < 0.001). Preop and postop 1^st^–7^th^–30^th^ day levels of endocan in the patient group were 379.8 (175.5–1573.3), 935.7 (132–1720.7), 654.9 (228.1–1412.3), and 732.3 (244.9–2061.2) pg/mL, respectively (p < 0.001). Typical ranges are 62–707 pg/mL for serum VEGF and 300–1200 pg/mL for serum endocan.

**Table 1:** Comparison of VEGF and endocan levels between patients in the preoperative period and controls.

	Patient group	Control group	p value
**VEGF (pg/mL)**	92.4 (11.2–338.4)	57.2 (0.1–311.9)	**0.001**
**Endocan (pg/mL)**	379.8 (175.5–1573.3)	562.7 (152.8–8383.7)	0.16

VEGF: vascular endothelial growth factor

Results presented as median values (range). Statistically significant p values are marked in bold.

**Table 2:** Comparison of VEGF levels, endocan levels, clinical characteristics, and laboratory findings between the patients grouped according to T scores.

	T1	T2	T3	p Value
**Preop VEGF (pg/mL)**	98.1 (11.2–338.4)	105.7 (44.3–298.6)	71.5 (13.9–244.7)	0.429
**Postop 1** **^st^** ** day VEGF (pg/mL)**	101.7 (45.7–293.5)	105.3 (32.4–163.1)	63 (0.7–124.3)	0.196
**Postop 7** **^th^** ** day VEGF (pg/mL)**	75 (0.1–249.1)	60.3 (0.1–353)	52.5 (1–99.9)	0.705
**Postop 30** **^th^** ** day VEGF (pg/mL)**	63.1 (0.1–353.6)	71.9 (31.4–194.1)	45.8 (1–72.1)	0.228
**Preop Endocan (pg/mL)**	331.1 (196.6–1039)	415 (175.4–1573.3)	368.5 (314.7–1074.3)	0.808
**Postop 1** **^st^** ** day Endocan (pg/ mL)**	736.7 (132–1720.7)	1024.3 (549.5–5001.9)	1084.6 (244.9–2855.4)	0.609
**Postop 7** **^th^** ** day Endocan (pg/ mL)**	597.5 (228.1–1765.9)	581.5 (273.6–5467)	1045 (539.8–1421.3)	0.102
**Postop 30** **^th^** ** day Endocan (pg/ mL)**	683.3 (271.6–2061.2)	825.9 (337.7–5193.9)	476.5 (198.2–2050.7)	0.272
**Age (years)**	61 (40–72)	60 (33–78)	58 (51–76)	0.732
**BMI (kg/m** **^2^** **)**	26 (22–31.1)	25.3 (20.2–31.6)	23.9 (21–28.4)	0.647
**Smoking (Pack–Year)**	30 (0–80)	15 (0–60)	30 (20–100)	0.103
**Preop WBC (10** **^9^** **/L)**	8.1 (3.3–12.8)	7.2 (4.3–9.4)	8.4 (5.8–11.1)	0.178
**Postop WBC (10** **^9^** **/L)**	10 (4.1–22.2)	9 (5.4–12.8)	9.2 (6.8–16.1)	0.644
**Preop NLR**	2.7 (1.1–8.9)	3.1 (1.4–7.9)	2.7 (1.7–3.9)	0.848
**Postop NLR**	3.5 (1–24.5)	3.6 (1.4–10.9)	3.2 (1.6–34)	0.981
**Preop CRP (mg/L)**	0.5 (0.2–2.3)	0.4 (0.1–3.1)	1.2 (0.2–5.3)	0.126
**Postop CRP (mg/L)**	0.9 (0.2–15.8)	0.7 (0.2–22.6)	5.3 (0.6–27)	0.131

VEGF: vascular endothelial growth factor, BMI: body mass index, WBC: white cell blood count, NLR: neutrophil to lymphocyte ratio, CRP: C-reactive protein

Results presented as median values (range). Statistically significant p values are marked in bold.

**Table 3:** Comparison of VEGF and endocan levels, clinical characteristics, and laboratory findings between patients grouped according to N status in NSCLC.

	N0	N1	p value
**Preop VEGF (pg/mL)**	84.8 (11.2–338.4)	100.4 (44.3–298.6)	0.438
**Postop 1** **^st^** ** day VEGF (pg/mL)**	89.5 (0.69–293.4)	105.6 (34.1–132.3)	0.797
**Postop 7** **^th^** ** day VEGF (pg/mL)**	61.2 (0.1–249.1)	57 (11.2–353)	0.719
**Postop 30** **^th^** ** day VEGF (pg/mL)**	58.6 (0.1–353.6)	65.6 (32.4–194.1)	0.671
**Preop Endocan (pg/mL)**	377.4 (175–1573.3)	410.3 (221–1595.5)	0.606
**Postop 1** **^st^** ** day Endocan (pg/mL)**	1003 (132–1720.7)	727.4 (279.6–5002)	0.854
**Postop 7** **^th^** ** day Endocan (pg/mL)**	662.2 (228–1765.9)	581.4 (331–1421.3)	0.894
**Postop 30** **^th^** ** day Endocan (pg/mL)**	679.4 (198–2061.2)	1280.4 (245–5194)	0.448
**Age (years)**	61(33–76)	55 (40–78)	0.083
**BMI (kg/m** **^2^** **)**	25.9 (21–31.6)	23.8 (20.1–29.3)	0.416
**Smoking (Pack–Year)**	30(0–100)	15 (0–30)	0.107
**Tumor Size (T cm)**	3 (0.5–6.9)	3.5 (3–4.5)	0.299
**Preop WBC (10** **^9^** **/L)**	8 (3.3–12.8)	7.2 (6.4–9.7)	0.768
**Postop WBC (10** **^9^** **/L)**	10 (5.4–22.2)	9.1 (6.2–15.8)	0.739
**Preop NLR**	2.76 (1–8.9)	2.6 (1.7–7.9)	0.883
**Postop NLR**	3.588 (1–34)	2.6 (2.3–11.2)	0.438
**Preop CRP (mg/L)**	0.65 (0.15–5.3)	0.76 (0.2–3)	0.685
**Postop CRP (mg/L)**	0.98 (0.19–27)	0.82 (0.31–22.6)	0.942

VEGF: vascular endothelial growth factor, BMI: body mass index, WBC: white cell blood count, NLR: neutrophil to lymphocyte ratio, CRP: C-reactive protein

Results presented as median values (range).

**Table 4:** Comparison of VEGF levels, endocan levels, clinical characteristics, and laboratory findings between patients grouped according to histopathological classification of tumor

	Adenocarcinoma (AC)	Squamous Cell Carcinoma (SCC)	Neuroendocrine Tumor (NET)	p Value
**Preop VEGF (pg/mL)**	85.4 (44.3–338.4)	114.6 (13.9–298.6)	90.2 (11.2–253.4)	0.835
**Postop 1** **^st^** ** day VEGF (pg/mL)**	103.6 (34.1–293.4)	88.9 (0.6–182.1)	65.5 (32.4–219.7)	0.459
**Postop 7** **^th^** ** day VEGF (pg/mL)**	62.1 (4.8–249.1)	87.3 (0.1–353)	36.9 (0.1–97.9)	**0.034**
	SCC vs NET			**0.038**
**Postop 30** **^th^** ** day VEGF (pg/mL)**	59 (6.5–346.8)	72 (1–194.1)	41.9 (0.1–353.6)	0.428
**Preop Endocan (pg/mL)**	435.8 (193.1–1595.5)	396.2 (266.3–1573.3)	304.5 (175.4–1039)	0.432
**Postop 1** **^st^** ** day Endocan (pg/ mL)**	1033.5 (132–1162.3)	702.8 (219–2967.4)	2091.6 (244.8–1720.7)	**0.041**
	SCC vs NET			**0.035**
**Postop 7** **^th^** ** day Endocan (pg/ mL)**	654.9 (331.2–1765.9)	585.2 (228–5467)	633.4 (273.6–1701.2)	0.635
**Postop 30** **^th^** ** day Endocan (pg/ mL)**	642.2 (198.1–5193.9)	662.6 (271.6–2061.2)	1011.3 (324.1–3264.4)	0.561
**Age (years)**	60 (49–72)	66.5 (50–78)	61 (33–76)	0.459
**BMI (kg/m** **^2^** **)**	25.6 (20.1–31.6)	25.5 (21.4–29)	25.5 (21.1–31.1)	0.949
**Smoking (Pack–Year)**	27.5 (0–100)	30 (10–80)	22.5 (0–45)	0.478
**Tumor Size (T cm)**	3.15 (0.5–6)	3.5 (2.2–5.5)	3.1 (0.8–6.9)	0.737
**Preop WBC (10** **^9^** **/L)**	8 (4.3–12.8)	8.3 (6–12.7)	7.3 (3–3.9)	0.365
**Postop WBC (10** **^9^** **/L)**	10 (5.4–22.2)	9.1 (6.2–15.8)	9.1 (4.1–12.8)	0.739
**Preop NLR**	2.8 (1.6–4.8)	2.8 (1.3–7.9)	2.3 (1.1–8.9)	0.788
**Postop NLR**	4.6 (1.7–34)	3.2 (1.4–10.9)	2.4 (1–7)	0.14
**Preop CRP (mg/L)**	0.64 (0.2–5.3)	0.95 (0.2–2.3)	0.28 (0.1–1.3)	**0.045**
	SCC vs NET			**0.043**
**Postop CRP (mg/L)**	0.76 (0.2–27)	1.5 (0.5–22.6)	0.86 (0.2–10.6)	0.23

VEGF: vascular endothelial growth factor, BMI: body mass index, WBC: white cell blood count, NLR: neutrophil to lymphocyte ratio, CRP: C-reactive protein

Results presented as median values (range). Statistically significant p values are marked in bold.

## Footnotes

1 American Lung Association (2022). State of Lung Cancer [online]. Website https://www.lung.org/getmedia/186786b6-18c3-46a9-a7e7-810f3ce4deda/SOLC-2023-Print-Report.pdf [accessed 11.06.2025]
